# Massively parallel transposon mutagenesis identifies temporally essential genes for biofilm formation in *Escherichia coli*

**DOI:** 10.1099/mgen.0.000673

**Published:** 2021-11-16

**Authors:** Emma R. Holden, Muhammad Yasir, A. Keith Turner, John Wain, Ian G. Charles, Mark A. Webber

**Affiliations:** ^1^​ Quadram Institute Bioscience, Norwich Research Park, Norwich, Norfolk NR4 7UQ, UK; ^2^​ Norwich Medical School, University of East Anglia, Norwich Research Park, Norwich, Norfolk NR4 7TJ, UK

**Keywords:** adhesion, functional genomics, TraDIS

## Abstract

Biofilms complete a life cycle where cells aggregate, grow and produce a structured community before dispersing to colonize new environments. Progression through this life cycle requires temporally controlled gene expression to maximize fitness at each stage. Previous studies have largely focused on identifying genes essential for the formation of a mature biofilm; here, we present an insight into the genes involved at different stages of biofilm formation. We used TraDIS-*Xpress*, a massively parallel transposon mutagenesis approach using transposon-located promoters to assay the impact of disruption or altered expression of all genes in the genome on biofilm formation. We identified 48 genes that affected the fitness of cells growing in a biofilm, including genes with known roles and those not previously implicated in biofilm formation. Regulation of type 1 fimbriae and motility were important at all time points, adhesion and motility were important for the early biofilm, whereas matrix production and purine biosynthesis were only important as the biofilm matured. We found strong temporal contributions to biofilm fitness for some genes, including some where expression changed between being beneficial or detrimental depending on the stage at which they are expressed, including *dksA* and *dsbA*. Novel genes implicated in biofilm formation included *zapE* and *truA* involved in cell division, *maoP* in chromosome organization, and *yigZ* and *ykgJ* of unknown function. This work provides new insights into the requirements for successful biofilm formation through the biofilm life cycle and demonstrates the importance of understanding expression and fitness through time.

## Data Summary

Sequence data supporting the analysis in this study has been deposited in ArrayExpress under the accession number E-MTAB-9873.

**Impact Statement**

Bacteria often exist in aggregated communities known as biofilms, the formation of which involves different sets of genes for different events, from colonization to maturation. The genetic basis for biofilm formation at different stages is not fully understood. Biofilms are a clinical concern due to their tolerance of high levels of antimicrobials, so understanding the development and key events in the biofilm life cycle will be key to finding new ways to prevent and manage bacterial infection and contamination. This study identified the genes and pathways that affect biofilm fitness through the biofilm’s development, using the recently developed transposon mutagenesis approach TraDIS-*Xpress*. Genes with roles in adhesion and motility were important for the fitness of the early biofilm, whereas matrix production and purine biosynthesis were only important as the biofilm matured. We also found roles for genes not previously described to affect biofilm formation. This work furthers our understanding of the requirements for biofilm formation at distinct stages of development. Additionally, this approach could be exploited to identify targets for anti-biofilm therapeutics or as biomarkers to identify biofilm infections, improving treatment efficacy.

## Introduction

Bacteria rarely exist planktonically outside of the laboratory and are usually found as part of structured, aggregated communities called biofilms [1]. Clinically, approximately 80 % of infections have been suggested to have a biofilm component [2], and biofilm-related infections are complicated by their intrinsic tolerance to antimicrobials, making infections difficult to treat and often persistent [3–6]. Cells within a biofilm grow more slowly than those in planktonic culture and this reduced level of metabolic activity has been associated with tolerance to antimicrobials, allowing biofilms to be typically 10–1000-fold less sensitive to antibiotics than corresponding strains in planktonic conditions [7, 8]. Aside from the clinical setting, there are many useful applications of biofilms, including wastewater treatment and bioprocessing [9]. Biofilms undergo a life cycle that commonly consists of initial attachment to a surface, growth and maturation of the biofilm over time with characteristic production of extracellular matrix components, followed by dispersal of planktonic cells to facilitate colonization of new surfaces [10]. The switch between planktonic and biofilm lifestyles is driven by environmental stimuli promoting large-scale changes in gene expression and regulation that are necessary to support the bacterial community through the life cycle, which is distinct from planktonic growth conditions. Expressing the right genes at the right time and place is critical for efficient production of a biofilm.

The main components of the biofilm extracellular matrix in *Escherichia coli* are the amyloid protein curli, the polysaccharide cellulose and extracellular DNA [11]. Genes involved in curli biosynthesis are transcribed by the divergent operons *csgBAC* and *csgDEFG*, with their expression regulated by CsgD [12]. Cellulose biosynthetic machinery is encoded by *bcsRQABZC* and *bcsEFG*, and its production is regulated by c-di-GMP [13]. Several genes are known to be involved in the regulation of matrix production, including *ompR* [14, 15], *cpxR* [14, 16, 17] and *rpoS* [18, 19], amongst others [20–22]. Extracellular DNA is also an important component of the biofilm matrix, and the addition of DNase has been shown to negatively affect the biomass of biofilms formed by *Pseudomonas aeruginosa* [23], *Bacillus cereus* [24] and a range of Gram-negative pathogens, including *E. coli* [25].

Many previous studies have focused on identifying the genes and pathways required for biofilm formation in *E. coli* in the mature biofilm. One assessed biofilm formation of all the mutants in the Keio collection [26, 27], another used a transcriptomic approach to identify genes with altered expression in biofilms over time [28], and DNA microarrays have also been used to link the presence of different genes with biofilm capacity in panels of isolates [29].

Large-scale transposon mutagenesis experiments represent another high-throughput, sensitive, whole-genome approach to link phenotype to genotype [30–32]. These methods make use of massive libraries of transposon mutants, where many independent mutants of each gene in the genome are represented in the pool. This provides great power in assaying the role of genes, and in a high-density library resolution is often high enough to make inferences about the intragenic essentiality of domains within proteins encoded by genes by analysing the fitness of multiple independent mutants within a gene. Transposon mutagenesis approaches, however, have been historically limited by an inability to assay essential genes within which transposon insertions are not viable. In order to provide information about these genes, we have recently developed TraDIS-*Xpress*. This method uses transposons containing an outward-facing inducible promoter. Addition of an inducer of the transposon-encoded promoter results in overexpression of genes downstream of transposon insertions, or repression of genes where the transposon is positioned downstream but in an antisense orientation. Therefore, we can assay the impact of altering expression of all genes (including those which cannot be inactivated), as well as capturing traditional essentiality measurements. We recently demonstrated the utility of this approach, and its ability to provide information about roles of essential genes in survival of drug exposure [33]. In this work, we sought to investigate biofilm formation using TraDIS-*Xpress* to get a more detailed view of important genes than possible in the previous studies described above. Predictions made by this approach were then tested in a range of assays that measure different aspects of biofilm formation using defined mutants from the Keio library [34], a collection of single knockout mutants in the same parent strain as the transposon mutant library.

This study identified 48 genes that were found to be important at different stages of biofilm formation by *E. coli *. By investigating the genes important across the biofilm life cycle, we were able to get a dynamic view of the main pathways with roles at different stages of biofilm development. Our findings reinforced the importance of adhesion, motility and matrix production in the biofilm, and revealed roles for genes not previously implicated in biofilm formation. This included genes involved in cell division, *zapE* [35] and *truA* [36], chromosome organization (*maoP*) [37], and *yigZ* and *ykgJ*, the functions of which have not been elucidated. We identified clear requirements for some pathways at specific points of the biofilm life cycle, furthering our understanding of how the fitness of cells in the biofilm is affected over time.

## Methods

### Transposon mutant library

The *E. coli* BW25113 transposon mutant library containing over 800 000 distinct mutants that was used in this study has recently been described by Yasir *et al*. [33]. The transposon used to construct this library incorporates an outward-transcribing IPTG-inducible promoter. This strain was chosen due to the high-quality transposon mutant library available, and because it is the parent strain for the Keio collection [34], an extensive library of single gene knockout mutants, which could be used to test defined mutants of genes where predictions were made from the TraDIS-*Xpress* data.

### Biofilm model conditions

The pooled mutant library was used to inoculate parallel cultures of 5 ml LB broth (without salt) with approximately 10^7^ cells. Cultures were grown in 6-well plates containing 40 sterile 5 mm glass beads per well (Sigma). Each experiment was set up with or without 1 mM IPTG for promoter induction. Plates were incubated at 30 °C with light shaking for 48 h. After 12, 24 and 48 h of incubation, a 2 ml planktonic sample was collected from each culture and 70 beads were taken to constitute the biofilm sample. Planktonic and biofilm samples were taken from the same well to match as closely as possible. Beads were washed twice in sterile 1x PBS and vortexed in tubes containing 1x PBS to resuspend cells from the biofilm. Both planktonic and biofilm samples were centrifuged at 2100 ***g*** to form pellets for DNA extraction. All conditions were run with two independent identical replicates.

### TraDIS*-Xpress* sequencing

Customized sequencing libraries were prepared to identify transposon insertions following the protocol described by Yasir *et al*. [33]. In short, DNA was extracted from pellets following the protocol described by Trampari *et al*. [38] and was fragmented using a MuSeek DNA fragment library preparation kit (ThermoFisher). Fragments containing transposons were amplified by PCR with Tn5-i5 and i7 primers customized to recognize the transposon and the MuSeek tagged ends of the DNA [33]. Fragments between 300 and 500 bp were size selected using AMPure beads (Beckman Coulter), and nucleotide sequences were generated using a NextSeq 500 and a NextSeq 500/550 High Output v2 kit (75 cycles) (Illumina). Between 1.7 and 26 million reads were obtained per condition.

### Informatics

Fastq files were aligned to the *E. coli* BW25113 reference genome (accession no. CP009273) using the BioTraDIS (version 1.4.3) software suite [39] using smalt (version 0.7.6). This generated plot files for visualization of the transposon insertion locus and frequency to compare planktonic and biofilm conditions. Conditions with and without IPTG were combined for initial analysis. Where a change in insert patterns was identified upstream or downstream of a coding sequence, the insert patterns from cultures grown with and without IPTG were manually visualized. This was to confirm an expression change was likely, and to determine whether there was a difference in distribution of reads with and without IPTG. Insertion frequencies per gene for each replicate were plotted against each other to determine the experimental error between replicates, as well as differences in insertion frequency between planktonic and biofilm conditions (Fig. S1, available with the online version of this article). The tradis_comparison.R command (also part of the BioTraDIS toolkit) was used to determine significant differences (*P*<0.05, after correction for false discovery) in insertion frequencies per gene between control and test conditions. Inserts predicted to only impact fitness of planktonic growth were excluded from further analysis. For all candidate loci, plot files generated by BioTraDIS were also examined manually in Artemis (version 17.0.1) [40] to confirm the results from these two approaches, as well as to identify regions where inserts were under differential selection but did not fall within coding regions of the genome.

### Validation experiments

The predicted impacts on biofilm formation of candidate genes were investigated further by testing both gene deletion mutants from the Keio collection (which contains two independent mutants for most genes in *E. coli* BW25113) for each gene [34]. These mutants were assessed in several assays relevant to different aspects of biofilm formation. Crystal violet assays, used to assess biofilm biomass production, were undertaken by inoculating 10^4^ of each mutant strain into 200 µl LB broth without salt in a 96-well polystyrene plate. After 48 h incubation at 30 °C, the culture was removed, wells were rinsed with water, and the residual biofilms were stained for 10 min with 200 µl 0.1 % crystal violet. The plate was then rinsed with water to remove the stain and 200 µl 70 % ethanol was added to the wells to solubilize the stained biofilm. The optical density was measured using a FLUOstar Omega plate reader (BMG Labtech) at 595 nm. Cell aggregation was measured by leaving bacterial cultures (normalized to an OD_600_ of 3.0) on an unagitated surface at room temperature. After 24 h, the supernatant of each culture was removed by pipetting, diluted in 1x PBS and measured in a plate reader at 600 nm. Biofilm matrix composition was investigated through spotting 10 µl of each mutant (representing 10^5^ c.f.u.) on agar supplemented with 40 µg Congo red ml^−1^ (Sigma) to examine curli production. Plates were incubated at 30 °C for 48 h and photographed to compare mutation strain biofilm composition to the wild-type. Adhesion and biofilm architecture were investigated under flow conditions for selected mutants using the Bioflux system. Flow cells were primed with LB broth without salt at 0.5 Pa and seeded with approximately 10^7^ cells. The plate was left at room temperature for 2.5 h to allow attachment, and subsequently incubated at 30 °C at a flow rate of 0.03 Pa. After 12, 24 and 48 h, biofilms were visualized with an inverted light microscope and representative images at ×10, ×20 and ×40 magnification were taken at three locations of the flow cell. Experiments were performed in duplicate.

## Results

### Confirmation of model efficacy

**Fig. 1.**
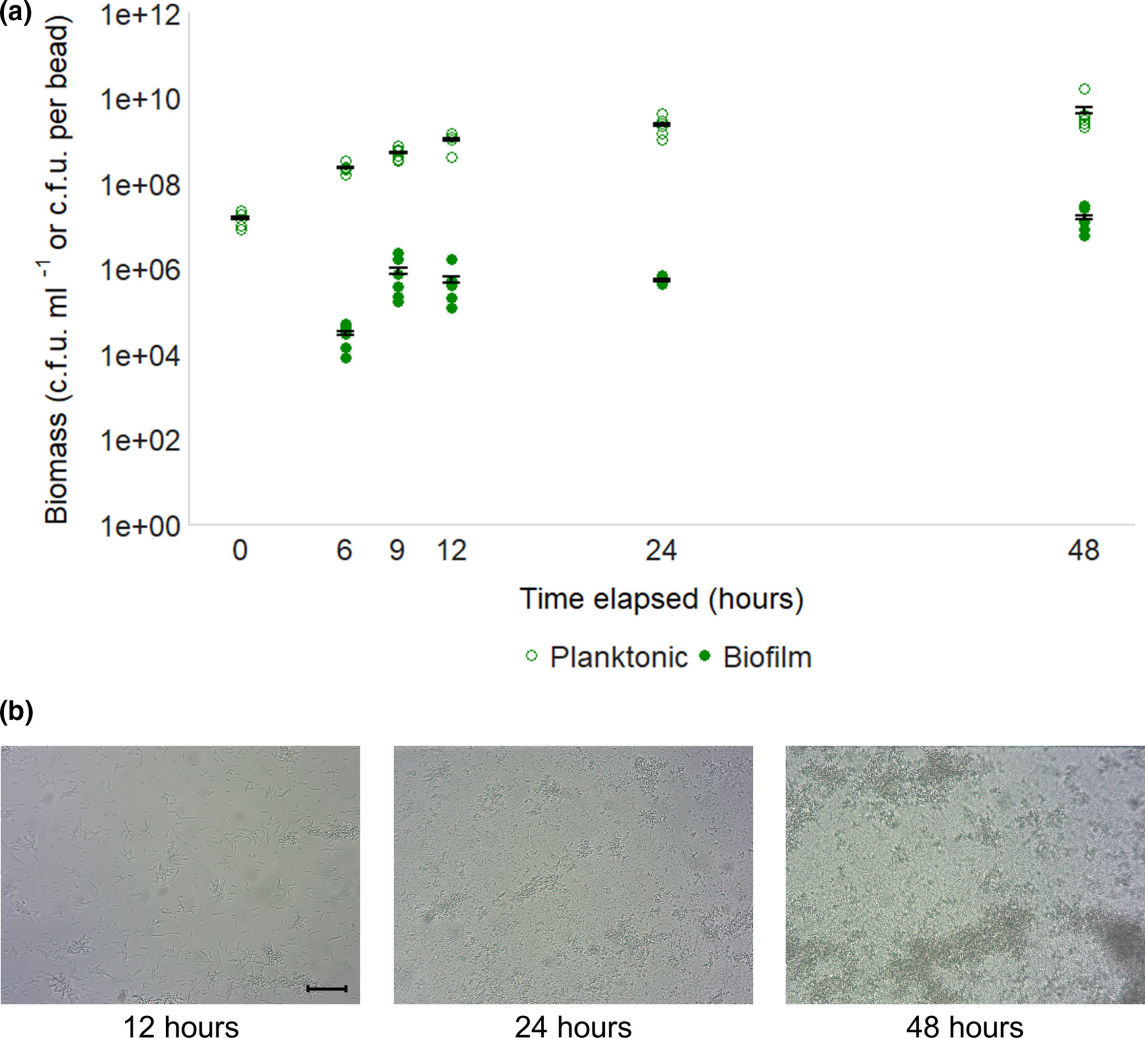
Biofilm formation of wild-type *E. coli* BW25113 on glass over time. (**a**) Numbers of c.f.u. of planktonic and biofilm samples harvested from the model at different time points through biofilm development. Planktonic samples are measured in c.f.u. ml^−1^ (culture), and biofilm samples are measured in c.f.u. of cells isolated from one glass bead. Points represent four independent replicates and error bars show 95 % confidence intervals where present. (**b**) Biofilms formed on glass under flow conditions after 12, 24 and 48 h growth. Images are representative of two independent replicates. Magnification ×20. Bar, 5 µm.

**Fig. 2.**
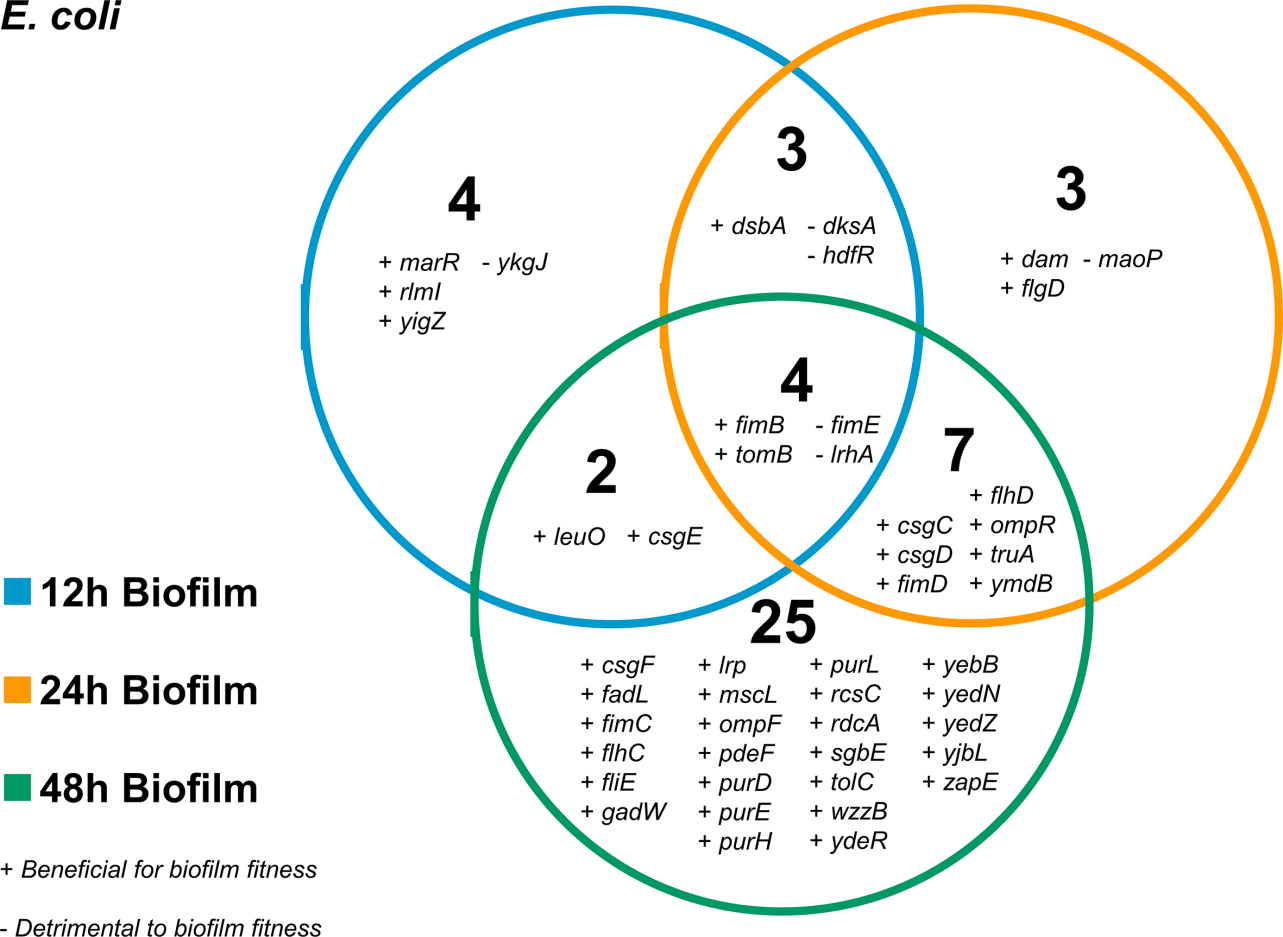
Genes involved in biofilm formation over time in *E. coli *. Plus symbols indicate genes that were beneficial for biofilm formation and minus symbols indicate genes that were detrimental to biofilm formation.

Wild-type *E. coli* BW25113 was grown on glass beads and harvested over time to investigate biofilm development after 12, 24 and 48 h (Fig. 1). The changes in biofilm c.f.u. (Fig. 1a) and architecture (Fig. 1b) after 12, 24 and 48 h growth show the development of the biofilm through time. A transposon mutant library containing approximately 800 000 unique mutants was then grown on glass beads and harvested at these time intervals. The genomic DNA obtained from biofilms and planktonic culture at each time point was analysed following the TraDIS-*Xpress* methodology to determine differences in gene essentiality and importance during biofilm formation over time. TraDIS-*Xpress* found 48 genes as candidates that considerably affected biofilm formation over time in *E. coli *: 42 were identified as being beneficial for biofilm fitness and 6 genes were predicted to be detrimental to the fitness of cells in the biofilm (Fig. 2, Table S1). The main pathways that were consistently important in the biofilm through all the time points included type 1 fimbriae, curli biosynthesis and regulation of flagella.

### Fimbriae expression and motility are important at all stages of biofilm formation

**Fig. 3.**
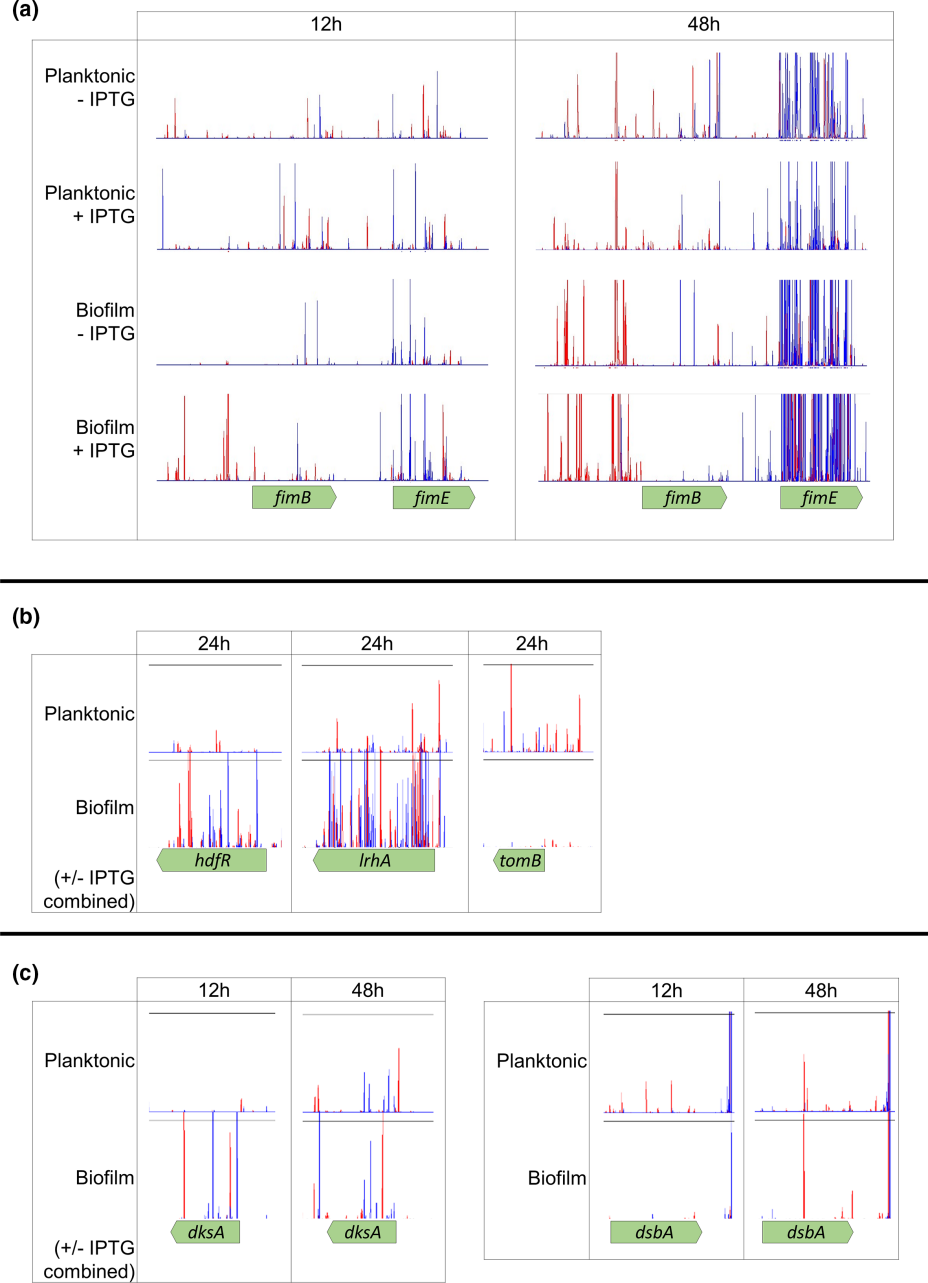
Transposon insertion sites and frequencies in planktonic and biofilm conditions, mapped to a reference genome and plotted with BioTraDIS in Artemis. The height of the peak can be used as a proxy for the mutant’s fitness in the condition. Red peaks indicate where the transposon-located promoter is facing left-to-right, and blue peaks show it facing right-to-left. (**a**) Insertion sites in and around *fimB* and *fimE* in planktonic and biofilm conditions after 12 and 48 h growth with and without promoter induction with IPTG. Leaky promoter expression is most likely responsible for the increased insertions upstream of *fimB* in conditions without IPTG. (**b**) Insertion sites in and around *hdfR*, *lrhA* and *tomB* in planktonic and biofilm conditions after 24 h growth. Conditions with and without IPTG have been combined. (**c**) Insertion sites in and around *dksA* and *dsbA* in planktonic and biofilm conditions after 12 and 48 h growth. Conditions with and without IPTG have been combined. For all plot files, one of two independent replicates is shown. The *y*-axes have been normalized for each locus to show relative differences in insert abundance between conditions.

**Fig. 4.**
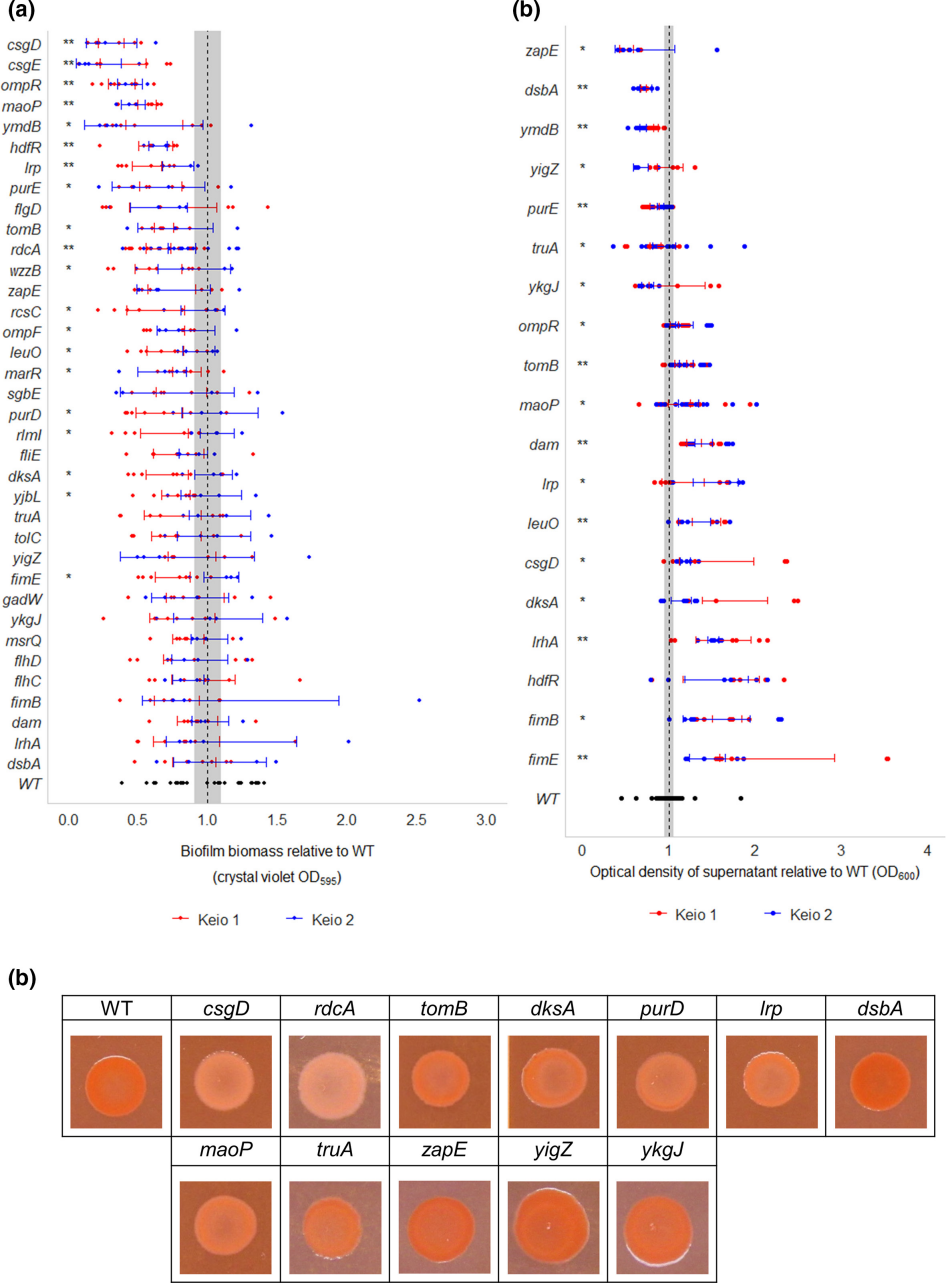
Phenotypic analysis of selected genes involved in biofilm formation. (**a**) Biofilm biomass of single knockout mutation strains relative to wild-type *E. coli *, measured by crystal violet staining. Two biological and a minimum of two technical replicates were performed for each mutation strain. (**b**) Cell aggregation of single knockout mutation strains relative to wild-type *E. coli *, measured by OD_600_ of the supernatant of unagitated cultures. Points show the OD_600_ of three independent replicates. For both graphs, coloured points/bars distinguish between the two Keio collection mutants of each gene. Error bars show 95 % confidence intervals, and the shaded area shows the 95% confidence interval of the wild-type. Single asterisks (*) represent a significant difference between one Keio mutant copy and the wild-type, and double asterisks (**) denote a significant difference between both Keio mutant copies and the wild-type (Welch’s *t*-test, *P*<0.05). (**c**) Colonies grown on agar supplemented with Congo red to compare curli biosynthesis between single knockout mutation strains and the wild-type. Images are representative of two biological and two technical replicates.

Only four genes were found to be important throughout 12, 24 and 48 h (Fig. 2). These included *fimB* and *fimE* involved in control of fimbriae expression, where deletion of *fimB* results in no fimbriated cells in a population, and deletion of *fimE* results in more fimbriated cells in a population relative to wild-type culture [41]. The recombinase gene *fimB*, which helps mediate both ‘ON-to-OFF’ and ‘OFF-to-ON’ switching of fimbriae expression, was beneficial for biofilm formation at all time points. There were fewer insertions within, and more insertions upstream of *fimB* in biofilm conditions compared to planktonic conditions at all time points. This suggests that *fimB* expression was beneficial throughout biofilm development (Fig. 3a). In contrast, inactivation of *fimE*, responsible for only ON-to-OFF fimbrial regulation [42], increased biofilm fitness at all time points. Initially, there were only slightly more *fimE* mutants in biofilm conditions compared to planktonic at 12 h, but this increased over time with a stark contrast seen between biofilm and planktonic conditions at the 24 and 48 h time points (Fig. 3a). Biofilm biomass was measured by growing knockout mutation strains in a 96-well plate for 48 h and staining the resulting biofilm with 0.1 % crystal violet. Cell aggregation was quantified by measuring the optical density of the supernatant of cultures left unagitated for 24 h. Deletion of *fimE* resulted in reduced biofilm biomass (Fig. 4a), contrary to the TraDIS-*Xpress* prediction, and both Δ*fimB* and Δ*fimE* mutation strains were deficient in cell aggregation (Fig. 4b). Together, the TraDIS-*Xpress* and phenotypic data suggest that the ability to regulate fimbriae expression in a phase-dependent manner is important for fitness of a biofilm, rather than being constrained in an ‘ON’ or ‘OFF’ state.

**Fig. 5.**
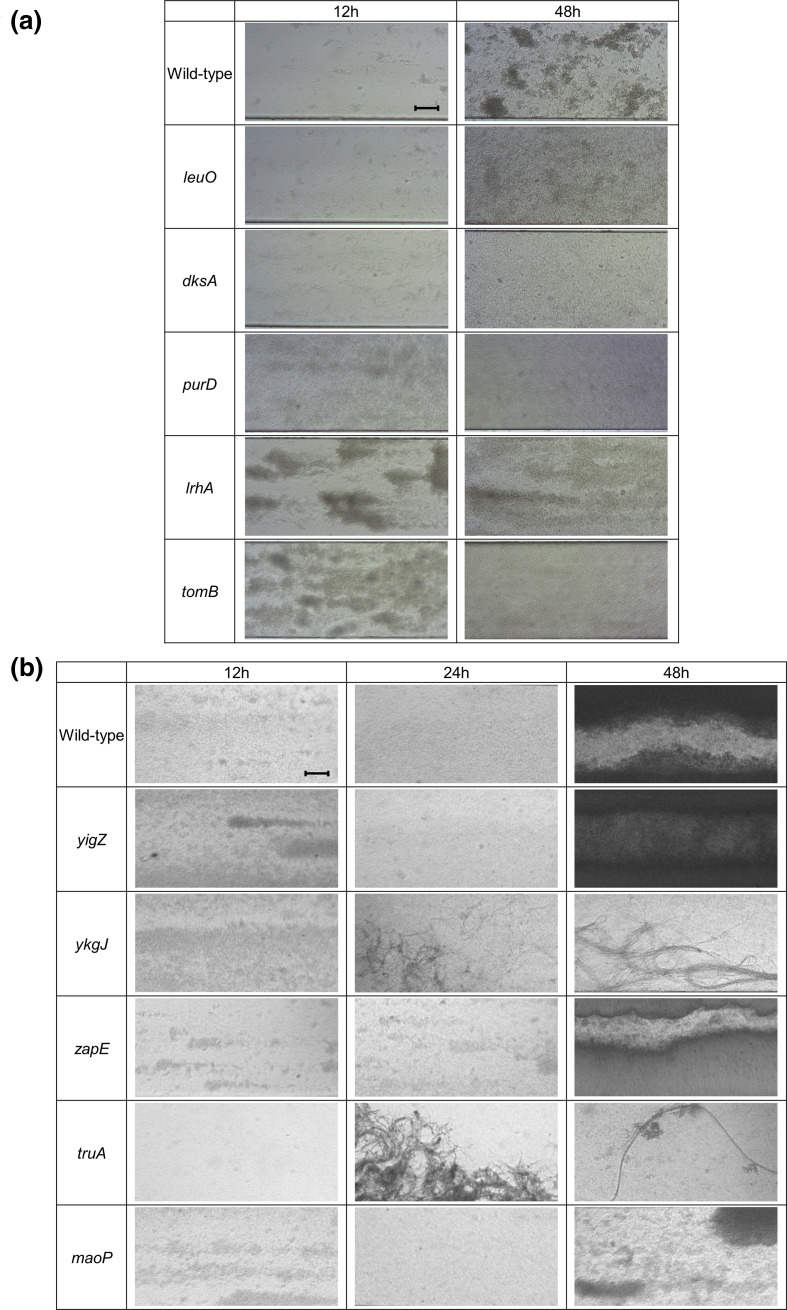
Biofilm formation of single knockout mutation strains on glass analysed under flow conditions after 12, 24 and 48 h growth. (**a**) Single knockout mutants selected for their effect on biofilm fitness. (**b**) Single knockout mutants of genes not previously described to affect biofilm formation, to the best of our knowledge. Magnification ×10. Images are representative of two independent replicates. Bar, 10 µm.

Disruption of *lrhA*, a regulator of motility and chemotaxis [43], was beneficial for biofilm formation at all time points (Fig. 3b). LrhA also has a role in type 1 fimbriae expression through activating expression of *fimE* [44], but in addition represses flagella-mediated motility. Analysis of the Δ*lrhA* biofilm showed initial formation of microcolonies occurred faster than the wild-type (Fig. 5a) but at later time points the biofilms formed by this mutation strain were less mature than seen with the wild-type. There was no significant change in biomass formed by this mutation strain (Fig. 4a) and the strain appeared less aggregative than the wild-type (Fig. 4b). These data suggest that inactivation of *lrhA* impacts both adhesion and aggregation differently at distinct stages of the biofilm life cycle, and may result in a benefit to early surface colonization but with a cost to later maturation.

Expression of the Hha toxin attenuator *tomB* was also found to be consistently important for biofilm formation at 12, 24 and 48 h (Fig. 3b). Consistent with this prediction, the Δ*tomB* mutation strain biofilm had reduced cell aggregation and curli biosynthesis, and reduced biofilm biomass (Fig. 4a–c). Under flow conditions, the Δ*tomB* mutation strain biofilm has a similar appearance to the Δ*lrhA* mutation strain biofilm, with microcolonies visible after 12 h growth, which disappeared over time (Fig. 5a).

### Regulatory genes are important in the early biofilm

In the early biofilm, after 12 h growth, only 13 genes were found to distinguish the planktonic and biofilm conditions. Of these, nine had roles in transcriptional regulation. The TraDIS-*Xpress* data indicated that inactivation of transcriptional factor *dksA* promoted biofilm formation at the 12 and 24 h time points but not in the mature biofilm (Fig. 3c). Supporting this, analysis of Δ*dksA* mutation strain biofilms under flow conditions showed an initial benefit with increased adhesion at both the 12 and 24 h time points, but reduced microcolony formation at the 48 h time point, suggesting *dksA* affects biofilm initiation (Fig. 5a). Inactivation of Δ*dksA* was also seen to reduce cell aggregation, curli biosynthesis and biofilm biomass (Fig. 4a–c). Expression of *hdfR*, a negative regulator of motility [45], was found to be detrimental to biofilm fitness in the early biofilm after 12 and 24 h growth (Fig. 3b), and Δ*hdfR* mutation strain biofilms had significantly reduced biomass (Fig. 4a). In addition, the stress response regulator *marR* [46] and the 23S rRNA methyltransferase *rlmI* [47] were both found to be beneficial for biofilm fitness at the 12 h time point only, and reduced biofilm biomass was found in the corresponding deletion mutation strains (Fig. 4a). These genes have both previously been implicated in biofilm formation [47–49], but the effect on early biofilm formation has not been described previously.

Two genes of unknown function, *yigZ* and *ykgJ*, were found to affect biofilm formation at 12 h. Fewer mutants were observed in *yigZ* in biofilm conditions relative to planktonic at 12 h, indicating its importance in early biofilm formation. We also saw that reduced expression of *ykgJ* was beneficial for biofilm formation, with more transposon insertions in an antisense orientation to *ykgJ* present in biofilm conditions relative to planktonic. Although there were no differences seen between the wild-type and *ykgJ* in biofilms grown under flow conditions for 12 h, differences became apparent at the 24 and 48 h time points, where the *ykgJ* mutation strain is significantly more filamented. For both *yigZ* and *ykgJ*, one mutant copy showed slightly increased aggregation relative to the wild-type (Fig. 4b), but there were no differences observed in biofilm biomass, curli biosynthesis or adhesion (Figs 4a, c and 5b).

### DNA housekeeping, adhesion and matrix production are important as biofilms mature

Two genes involved in DNA housekeeping were found to be involved in biofilm development after 24 h growth. This included *dam*, encoding DNA methyltransferase [50], insertional activation of which was not tolerated in the 24 h biofilm, with Δ*dam* mutation strains defective in aggregation compared to the wild-type (Fig. 4b). Also, inactivation of *maoP*, involved in Ori macrodomain organization [37], was predicted to confer a fitness advantage in the 24 h biofilm compared to the planktonic condition. TraDIS-*Xpress* data showed more reads mapped to *maoP* in the biofilm conditions compared to the planktonic at 24 h, suggesting loss of this gene was beneficial. Phenotypic analysis of the Δ*maoP* mutation strain biofilm did demonstrate a phenotype although in opposition to the prediction, *maoP* mutation strains were significantly deficient in biofilm biomass production, curli biosynthesis and one mutant displayed reduced aggregation (Fig. 4a, c). After 48 h growth under flow conditions, Δ*maoP* mutation strain biofilm was considerably less dense than the wild-type (Fig. 5b).

There were fewer insertions detected within *dsbA* (encoding disulphide oxidoreductase) [51] in biofilms grown for 12 and 24 h relative to planktonic culture (Fig. 3c). The role of *dsbA* in adhesion to abiotic surfaces and epithelial cells has previously been suggested [51, 52]. Phenotypic validation of the Δ*dsbA* mutation strain showed a red, dry and rough (*rdar*) phenotype on Congo red plates (Fig. 4c), indicative of increased curli biosynthesis. Cell aggregation in the Δ*dsbA* mutation strain was significantly higher compared to the wild-type, implying a role of *dsbA* in inhibiting cell–cell aggregation. Our data showed that *dsbA* is important in the early biofilm, but its deletion appears to be beneficial to the formation of a mature biofilm, according to the Congo red and aggregation data.

### Mature biofilm requires purine biosynthesis, matrix production, motility and solute transport

There were 38 genes found to be important for fitness of the mature biofilm after 48 h growth, and 25 of these genes were identified to affect fitness at this time point only. The major pathway implicated in biofilm development at 48 h was purine ribonucleotide biosynthesis, with four genes, *purD*, *purH*, *purL* and *purE* [53], found to be essential at this time point only. TraDIS-*Xpress* did not identify mutants in any of these genes in biofilms sampled at 48 h, whereas several reads mapped to these loci under planktonic conditions, as well as under both biofilm and planktonic conditions earlier at 12 and 24 h. Visualization of a Δ*purD* mutation strain biofilm under flow conditions saw poor biofilm formation and no microcolony formation at any time compared to the wild-type (Fig. 5a). Additionally, Δ*purD* and Δ*purE* mutation strains were deficient in biofilm biomass production, curli biosynthesis, and Δ*purE* also showed increased cell aggregation (Fig. 4a–c), confirming an important role for purine biosynthesis in matrix production and curli biosynthesis in the mature biofilm.

Two genes involved in cell division, *zapE* [35] and *truA* [36], were identified as important in the 48 h biofilm. No mutants were seen within *zapE* in biofilms grown for 48 h, suggesting its essentiality for biofilm development at this stage. This was, however, not reflected in the phenotype of the defined Keio deletion mutants tested, with no changes observed in biofilm biomass or curli biosynthesis, and increased aggregation seen in Δ*zapE* mutation strains relative to the wild-type (Fig. 4a–c). A *zapE* mutation strain did have considerably reduced adhesion after 12 h growth under flow conditions, relative to the wild-type (Fig. 5b). The pseudouridine synthase *truA* [54] was found to be essential in the mature biofilm grown for 24 and 48 h, and when grown independently under flow conditions, Δ*truA* mutation strain cells were extremely filamented in biofilms (Fig. 5b).

The flagella master regulatory system *flhDC* was identified as important in the mature biofilm. Biofilms sampled after 48 h saw fewer *flhC* mutants, while insertions interpreted as over-expressing *flhD* increased in numbers both at the 24 and 48 h time points, compared to planktonic conditions. No mutants in *flgD* and *fliE*, encoding flagellar filament proteins, were identified at 24 and 48 h, respectively. It has previously been shown that motility is important for initial biofilm formation [55, 56], but this may not relate to biomass formation where no differences were seen for Δ*flhD*, Δ*flhC*, Δ*fliE* and Δ*flgD* mutants.

Various pleiotropic transcriptional regulators were also important in the mature biofilm. This included the H-NS antagonist *leuO* [57]. Increased insertions upstream of *leuO* under biofilm conditions after 12 h growth, as well as no *leuO* mutants in 48 h biofilms, indicated it was beneficial to biofilm formation. A Δ*leuO* mutation strain did not aggregate as well as the wild-type, and one Δ*leuO* mutation strain had reduced biofilm biomass (Fig. 4a, b). The Δ*leuO* mutation strain biofilm under flow conditions demonstrated an inability to form microcolonies after 48 h growth (Fig. 5a). There were also fewer mutants within *lrp*, the leucine-responsive global regulator [58], and *gadW*, a transcriptional regulator responsible for survival under acid stress [59], in the 48 h biofilm compared to the planktonic condition, indicating their importance in the mature biofilm. Reduced biofilm biomass, aggregation and curli biosynthesis were observed for one copy of Δ*lrp*, but no differences in biofilm formation or aggregation were seen for Δ*gadW* mutation strain biofilms (Fig. 4a–c).

## Discussion

**Fig. 6.**
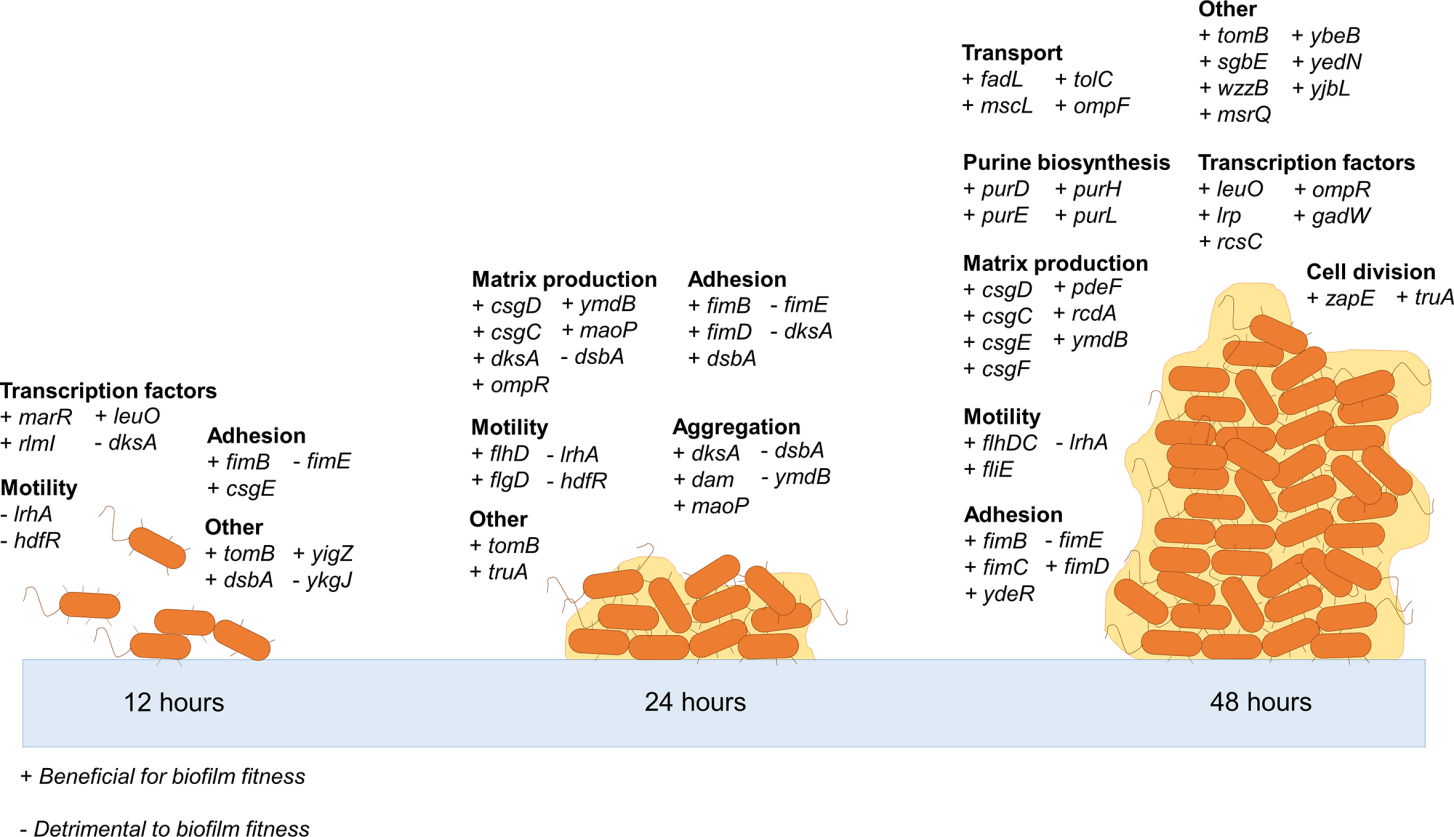
Summary of genes important for biofilm formation by *E. coli* at different stages of development.

We have characterized the essential genome of *E. coli* biofilms across the life cycle(Fig. 6). The identification of genes and pathways already described to be involved in biofilm formation validates the efficacy of this experimental model and shows how assessing many mutants in parallel can identify many genes involved in a phenotype using a single set of experiments. Different genes showed importance at different stages of biofilm; the early biofilm established 12 h after inoculation was characterized by genes involved in adhesion. The 24 h biofilm required both adhesion and matrix production, and after 48 h genes involved in matrix production, cell division and purine biosynthesis were beneficial to biofilm fitness. In concordance with previous work identifying genes whose importance varies with time in the *E. coli* biofilm, we also reported that control of fimbriae expression and motility remained important at each stage of the biofilm life cycle rather than just being involved in initial attachment [28].

TraDIS-*Xpress* was able to identify several genes not previously reported to be involved in biofilm formation, including *yigZ*, *ykgJ*, *zapE*, *maoP* and *truA*. The TraDIS-*Xpress* data predicted that expression of *maoP* was detrimental to the fitness of biofilms grown for 24 h, but a Δ*maoP* mutation strain biofilm had reduced biofilm biomass and reduced curli biosynthesis compared to the wild-type. A homologue to *maoP* in *Yersinia pestis* was identified as having a role in adhesion and may positively regulate adhesin expression [60]. It is unclear why the defined mutants made less biofilm that the wild-type when TraDIS-*Xpress* predicted expression of *maoP* was detrimental to biofilm formation. Chromosomal organization of the Ori macrodomain requires both *maoP* and *maoS* [37], and it may be that deletion of *maoP* affects the interplay between these two genes. Further investigation into how chromosomal macrodomain organization affects biofilm formation is warranted. The importance of cell division in the mature biofilm was shown by our observation of fewer *zapE* and *truA* mutants surviving in biofilm conditions compared to planktonic conditions. We found reduced adhesion in the Δ*zapE* mutation strain biofilm and increased filamentation in the Δ*truA* mutation strain biofilm. ZapE has been found to be required for growth under low oxygen conditions as well as having a role in cell division [35], and this may be relevant for why its expression was beneficial for cells within a submerged biofilm. Deletion of *truA* has previously been reported to result in filament formation and reduced cell division [36], and increased expression of *truA* was seen to benefit intracellular survival and survival under oxidative stress conditions [61]. Deletion of *ykgJ* was also found to cause filament formation in biofilms grown for 24 and 48 h, which suggests a role in cell division for this gene. Filamentation has previously been suggested to provide a competitive advantage in adhesion and early biofilm formation, but filamented cells were outcompeted as the biofilm matured [62].

This study has highlighted the benefit of close temporal gene regulation in the biofilm, where the expression of certain genes may only be required at one stage or can even have a different effect on biofilm fitness at different stages of the biofilm life cycle. For example, we found that *dsbA* was important for the early biofilm, and previous work has shown DsbA-DsbB facilitates export and assembly of various adhesins by acting as a chaperone [52].We found that *dsbA* deletion resulted in increased curli expression and increased aggregation. Expression of *dsbA* has been previously found to result in repression of the curli regulator *csgD* and curli subunit *csgA*, essential for optimal fitness of the mature biofilm [63]. Conversely, we found that disruption of the transcription factor *dksA* was beneficial in the early biofilm, whilst a *dksA* knockout strain biofilm had reduced biofilm biomass, reduced curli biosynthesis and reduced aggregation. The effect of *dksA* expression of biofilm formation has been extensively studied and it is known that the deletion of *dksA* increases fimbriae-dependent adhesion, but reduces motility [64] and curli production [32, 65, 66]. Again, these data show differential expression of important genes at different stages of the biofilm life cycle is essential for optimizing biofilm fitness.

Purine biosynthesis was found to be important in the mature biofilm, through the essentiality of *purD*, *purE*, *purL* and *purH* in biofilms grown for 48 h. Similar findings have previously been described in another transposon mutagenesis experiment in uropathogenic *E. coli* [32]. Inactivation of purine biosynthetic genes was also found to impair biofilm formation in *B. cereus *, but this was thought to be due to reduced extracellular DNA in the biofilm matrix [24]. Extracellular DNA is thought to aid adhesion and has been found to be important in the biofilms of a wide range of bacterial species [23, 25]. Our data suggest the importance is in the mature biofilm rather than initial adhesion. A relationship between both purine and pyrimidine biosynthesis and curli production in the biofilm has been reported [32, 65, 67] and curli biosynthesis in a *purL* mutation strain was reported to be abrogated through addition of inosine, which is involved in the *de novo* purine biosynthetic pathway [68]. This suggests that nucleotide production itself, rather than the regulatory effects of the genes involved, affects curli biosynthesis, supporting one hypothesis that disruption of the purine biosynthetic pathway may directly result in a reduction of c-di-GMP. In support of this, we identified two genes involved in c-di-GMP metabolism, *rcdA* and *pdeF* [69], to be important for biofilm formation at 48 h. The effects of c-di-GMP on biofilm biomass production and curli biosynthesis have been thoroughly described [32, 69]. Quantification of intracellular c-di-GMP or further investigation of other c-di-GMP-dependent pathways in these mutants would uncover the relationship between these pathways and biofilm formation.

The relationship between motility and biofilm formation is complex. Although it is widely understood that motility is crucial for initial adhesion [55, 56], there is also an inverse relationship between motility and expression of matrix components; when biofilm matrix production is induced, motility is repressed in a motile-to-sessile lifestyle transition [66, 70, 71]. We found that insertional inactivation of negative motility regulators *lrhA* and *hdfR* improved biofilm fitness according to the TraDIS-*Xpress* data. Interestingly, our data found an important role for structural flagella components only in the mature biofilm, this observation is supported by previous work that found expression of flagella is important at all stages of the developing biofilm [28]. Previous work has suggested that flagella filaments are important for initial attachment and adhesion [72]; however, we did not find this to be the case, with genes encoding flagella filaments only appearing to contribute to biofilm fitness in the mature biofilm. It appears that maintaining the ability to flexibly regulate production of flagella and motility, rather than their fixed expression or absence, is important for optimal biofilm fitness of a population throughout biofilm development.

Analysis of biofilms under flow conditions found that Δ*lrhA* and Δ*tomB* mutation strain biofilms had a similar appearance after 12 h growth, with microcolonies visible that disappeared over time. The similarities in phenotypes could indicate both genes influence biofilms in a similar manner. The role of *lrhA* in motility regulation has been well documented [43, 44, 73], and expression of *tomB* has been seen to reduce motility through repression of *fliA* [74]. Although Δ*lrhA* and Δ*tomB* deletion mutation strains shared many similar phenotypes, TraDIS-*Xpress* data predicted that *tomB* was beneficial and *lrhA* was detrimental to biofilm development at 12, 24 and 48 h. Therefore, these genes may regulate the same pathways but in different ways. Previous studies on Δ*lrhA* mutation strain biofilms have reported increased adhesion, aggregation and biomass compared to the wild-type [44]. This supports the findings from the TraDIS-*Xpress* data, showing inactivation of *lrhA* was beneficial for biofilm fitness throughout biofilm development. This may be due to reduced induction of *fimE* by LrhA [44], thereby allowing expression of type 1 fimbriae to facilitate adhesion. We have already described how expression of both *fimB* and *fimE* is necessary for optimal fitness of the mature biofilm, and the effect of *lrhA* on biofilm formation correlates with these findings, with reduced aggregation in Δ*lrhA* biofilms after 24 h (also seen in *fimB* and *fimE* mutation strains) and no microcolony formation under flow conditions at 24 and 48 h. The importance of *lrhA* to biofilm formation clearly appears to be time dependent, with the most important role in early events. Studies on the effect of *tomB* on biofilm formation have focused on its toxin–antitoxin relationship with *hha*, which has been found to reduce expression of fimbrial subunit *fimA* and activate prophage lytic genes causing cell death [75]. Deletion of *hha* was found to reduce motility through *flhDC* and increase curli production through *csgD* [76]. We found no obvious benefit to biofilm fitness with insertional inactivation of *hha*, but this may not be visible in our data due to these mutation strains having a functional copy of *tomB*, which would mask impacts from loss of *hha.*

Previous genome-wide screens on *E. coli* biofilm formation have identified many of the same genes as this study [26, 30, 32]. The TraDIS-*Xpress* technology used here differentiates this work, as we are able to predict the effect of changes in gene expression and gene essentiality over time. We found the overexpression of three genes and reduced expression of one gene was beneficial for biofilm fitness. Differences between this work and previous studies may reflect differences in experimental conditions, which can affect biofilm formation [77], and may also reflect the high sensitivity of transposon mutagenesis approaches where genes with small impacts on fitness can be identified in massive competition experiments. Most of the defined mutants tested here did have a phenotype in one or more of the validation experiments we used (Table S1) but some did not. Whole-gene-knockout mutants differ from transposon insertion mutants, with an insertion on average every 6 bp, the mutant library used here gives an in-depth screen of exactly which regions of the genes in question are important for a given phenotype [33]. In addition, the TraDIS-*Xpress* experiments involved competition of each mutant against the rest of the pool, this is very sensitive to changes in fitness. Whilst we chose a set of important biofilm-associated phenotypes for validation of our candidate important genes using defined mutants, these are inevitably somewhat crude and cannot replicate the competition happening within the biofilms in the main experiments. It is likely we failed to identify the basis for a phenotypic impact of some of our candidate mutants in our limited validation conditions with whole-gene inactivation mutants.

Various genes were expected to be identified by the model to confirm its efficacy, such as genes involved in curli biosynthesis; however, there were some genes that were not detected by TraDIS-*Xpress* that are known to affect biofilm formation. Although many genes involved in curli biosynthesis were identified by our model, the gene encoding the main curli subunit, *csgA*, was not detected. This is likely to be because TraDIS-*Xpress* experiments use a mutant library pool, where CsgA produced by the surrounding population will complement any Δ*csgA* mutants [78]. Although this may be a potential limitation for studying a gene’s role in biofilm formation, it is more representative of intercellular interactions in a non-clonal multispecies biofilm found outside the laboratory. We also did not identify antigen 43 (*agn43/flu*) as important for biofilm formation, despite its strong role in aggregation and adhesion [79, 80]. Previous work found antigen 43 was important for biofilm formation in glucose-minimal media, but not LB [80]. This justifies the need for more genome-wide studies analysing a wide range of environmental conditions, strains and species, abiotic and biotic surfaces, to provide a wider list of conditionally essential genes for biofilm formation shared amongst important human pathogens. As well as temporal changes in gene expression, spatial changes have been shown to affect biofilm development [81]. Integration of the spatial component into this model, to assay how gene expression throughout the biofilm over time affects biofilm fitness, would be the next logical step in furthering our understanding of biofilm development.

This study has revealed important time-specific roles for known and identified novel genes with roles in biofilm formation. We reveal some pathways have a more important role in the mature biofilm than previously appreciated and identify genes with time-dependent conditional essentiality within the biofilm. We also identify potential new candidate genes essential for biofilm formation, which could be targeted for novel anti-biofilm therapies. Further work using high-density transposon mutant libraries across time and in different conditions is likely to further our understanding of biofilm biology.

## Supplementary Data

Supplementary material 1

## References

[1] Berlanga M, Guerrero R (2016). Living together in biofilms: the microbial cell factory and its biotechnological implications. Microb Cell Fact.

[2] Bjarnsholt T, Buhlin K, Dufrêne YF, Gomelsky M, Moroni A (2018). Biofilm formation – what we can learn from recent developments. J Intern Med.

[3] Gbejuade HO, Lovering AM, Webb JC (2015). The role of microbial biofilms in prosthetic joint infections. Acta Orthop.

[4] Davis SC, Martinez L, Kirsner R (2006). The diabetic foot: the importance of biofilms and wound bed preparation. Curr Diab Rep.

[5] Vestby LK, Grønseth T, Simm R, Nesse LL (2020). Bacterial biofilm and its role in the pathogenesis of disease. Antibiotics.

[6] Wang H, Tay M, Palmer J, Flint S (2017). Biofilm formation of *Yersinia enterocolitica* and its persistence following treatment with different sanitation agents. Food Control.

[7] Mah TF, Pitts B, Pellock B, Walker GC, Stewart PS (2003). A genetic basis for *Pseudomonas aeruginosa* biofilm antibiotic resistance. Nature.

[8] Hoyle BD, Costerton JW (1991). Bacterial resistance to antibiotics: the role of biofilms. Prog Drug Res.

[9] Flemming H-C, Wingender J, Szewzyk U, Steinberg P, Rice SA (2016). Biofilms: an emergent form of bacterial life. Nat Rev Microbiol.

[10] Kostakioti M, Hadjifrangiskou M, Hultgren SJ (2013). Bacterial biofilms: development, dispersal, and therapeutic strategies in the dawn of the postantibiotic era. Cold Spring Harb Perspect Med.

[11] Flemming H-C, Wingender J (2010). The biofilm matrix. Nat Rev Microbiol.

[12] Barnhart MM, Chapman MR (2006). Curli biogenesis and function. Annu Rev Microbiol.

[13] Serra DO, Hengge R, Cohen E, Merzendorfer H (2019). Extracellular Sugar-Based Biopolymers Matrices.

[14] Jubelin G, Vianney A, Beloin C, Ghigo J-M, Lazzaroni J-C (2005). CpxR/OmpR interplay regulates curli gene expression in response to osmolarity in *Escherichia coli*. J Bacteriol.

[15] Vidal O, Longin R, Prigent-Combaret C, Dorel C, Hooreman M (1998). Isolation of an *Escherichia coli* K-12 mutant strain able to form biofilms on inert surfaces: involvement of a new OmpR allele that increases curli expression. J Bacteriol.

[16] Dorel C, Vidal O, Prigent-Combaret C, Vallet I, Lejeune P (1999). Involvement of the Cpx signal transduction pathway of *E. coli* in biofilm formation. FEMS Microbiol Lett.

[17] Otto K, Silhavy TJ (2002). Surface sensing and adhesion of *Escherichia coli* controlled by the Cpx-signaling pathway. Proc Natl Acad Sci USA.

[18] Adams JL, McLean RJ (1999). Impact of *rpoS* deletion on *Escherichia coli* biofilms. Appl Environ Microbiol.

[19] Corona-Izquierdo FP, Membrillo-Hernández J (2002). A mutation in *rpoS* enhances biofilm formation in *Escherichia coli* during exponential phase of growth. FEMS Microbiol Lett.

[20] Gerstel U, Park C, Römling U (2003). Complex regulation of *csgD* promoter activity by global regulatory proteins. Mol Microbiol.

[21] Gerstel U, Römling U (2003). The *csgD* promoter, a control unit for biofilm formation in *Salmonella typhimurium*. Res Microbiol.

[22] Amores GR, de Las Heras A, Sanches-Medeiros A, Elfick A, Silva-Rocha R (2017). Systematic identification of novel regulatory interactions controlling biofilm formation in the bacterium *Escherichia coli*. Sci Rep.

[23] Whitchurch CB, Tolker-Nielsen T, Ragas PC, Mattick JS (2002). Extracellular DNA required for bacterial biofilm formation. Science.

[24] Vilain S, Pretorius JM, Theron J, Brözel VS (2009). DNA as an adhesin: *Bacillus cereus* requires extracellular DNA to form biofilms. Appl Environ Microbiol.

[25] Tetz GV, Artemenko NK, Tetz VV (2009). Effect of DNase and antibiotics on biofilm characteristics. Antimicrob Agents Chemother.

[26] Niba ETE, Naka Y, Nagase M, Mori H, Kitakawa M (2007). A genome-wide approach to identify the genes involved in biofilm formation in *E. coli*. DNA Res.

[27] Aedo SJ, Ma HR, Brynildsen MP (2019). Checks and balances with use of the Keio collection for phenotype testing. Methods Mol Biol.

[28] Domka J, Lee J, Bansal T, Wood TK (2007). Temporal gene-expression in *Escherichia coli* K-12 biofilms. Environ Microbiol.

[29] Schembri MA, Kjaergaard K, Klemm P (2003). Global gene expression in *Escherichia coli* biofilms. Mol Microbiol.

[30] Puttamreddy S, Cornick NA, Minion FC (2010). Genome-wide transposon mutagenesis reveals a role for pO157 genes in biofilm development in *Escherichia coli* O157:H7 EDL933. Infect Immun.

[31] Goh KGK, Phan M-D, Forde BM, Chong TM, Yin W-F (2017). Genome-wide discovery of genes required for capsule production by uropathogenic *Escherichia coli*. mBio.

[32] Nhu NTK, Phan M-D, Peters KM, Lo AW, Forde BM (2018). Discovery of new genes involved in curli production by a uropathogenic *Escherichia coli* strain from the highly virulent O45:K1:H7 lineage. mBio.

[33] Yasir M, Turner AK, Bastkowski S, Baker D, Page AJ (2020). TraDIS-Xpress: a high-resolution whole-genome assay identifies novel mechanisms of triclosan action and resistance. Genome Res.

[34] Baba T, Ara T, Hasegawa M, Takai Y, Okumura Y (2006). Construction of *Escherichia coli* K-12 in-frame, single-gene knockout mutants: the Keio collection. Mol Syst Biol.

[35] Marteyn BS, Karimova G, Fenton AK, Gazi AD, West N (2014). ZapE is a novel cell division protein interacting with FtsZ and modulating the z-ring dynamics. mBio.

[36] Tsui HC, Arps PJ, Connolly DM, Winkler ME (1991). Absence of hisT-mediated tRNA pseudouridylation results in a uracil requirement that interferes with *Escherichia coli* K-12 cell division. J Bacteriol.

[37] Valens M, Thiel A, Boccard F (2016). The MaoP/*maoS* site-specific system organizes the Ori region of the *E. coli* chromosome into a macrodomain. PLoS Genet.

[38] Trampari E, Holden ER, Wickham GJ, Ravi A, de Oliveira Martins L (2021). Exposure of *Salmonella* biofilms to antibiotic concentrations rapidly selects resistance with collateral tradeoffs. NPJ Biofilms Microbiomes.

[39] Barquist L, Mayho M, Cummins C, Cain AK, Boinett CJ (2016). The TraDIS toolkit: sequencing and analysis for dense transposon mutant libraries. Bioinformatics.

[40] Carver T, Harris SR, Berriman M, Parkhill J, McQuillan JA (2012). Artemis: an integrated platform for visualization and analysis of high-throughput sequence-based experimental data. Bioinformatics.

[41] McClain MS, Blomfield IC, Eberhardt KJ, Eisenstein BI (1993). Inversion-independent phase variation of type 1 fimbriae in *Escherichia coli*. J Bacteriol.

[42] Klemm P (1986). Two regulatory fim genes, fimB and fimE, control the phase variation of type 1 fimbriae in *Escherichia coli*. EMBO J.

[43] Lehnen D, Blumer C, Polen T, Wackwitz B, Wendisch VF (2002). LrhA as a new transcriptional key regulator of flagella, motility and chemotaxis genes in *Escherichia coli*. Mol Microbiol.

[44] Blumer C, Kleefeld A, Lehnen D, Heintz M, Dobrindt U (2005). Regulation of type 1 fimbriae synthesis and biofilm formation by the transcriptional regulator LrhA of *Escherichia coli*. Microbiology.

[45] Ko M, Park C (2000). H-NS-dependent regulation of flagellar synthesis is mediated by a LysR family protein. J Bacteriol.

[46] Alekshun MN, Levy SB (1999). Alteration of the repressor activity of MarR, the negative regulator of the *Escherichia coli marRAB* locus, by multiple chemicals *in vitro*. J Bacteriol.

[47] Herzberg M, Kaye IK, Peti W, Wood TK (2006). YdgG (TqsA) controls biofilm formation in *Escherichia coli* K-12 through autoinducer 2 transport. J Bacteriol.

[48] Holden ER, Webber MA (2020). MarA, RamA, and SoxS as mediators of the stress response: survival at a cost. Front Microbiol.

[49] Kettles RA, Tschowri N, Lyons KJ, Sharma P, Hengge R (2019). The *Escherichia coli* MarA protein regulates the *ycgZ-ymgABC* operon to inhibit biofilm formation. Mol Microbiol.

[50] Szyf M, Avraham-Haetzni K, Reifman A, Shlomai J, Kaplan F (1984). DNA methylation pattern is determined by the intracellular level of the methylase. Proc Natl Acad Sci USA.

[51] Lee Y, Kim Y, Yeom S, Kim S, Park S (2008). The role of disulfide bond isomerase A (DsbA) of *Escherichia coli* O157:H7 in biofilm formation and virulence. FEMS Microbiol Lett.

[52] Bringer MA, Rolhion N, Glasser AL, Darfeuille-Michaud A (2007). The oxidoreductase DsbA plays a key role in the ability of the Crohn’s disease-associated adherent-invasive *Escherichia coli* strain LF82 to resist macrophage killing. J Bacteriol.

[53] Zhang Y, Morar M, Ealick SE (2008). Structural biology of the purine biosynthetic pathway. Cell Mol Life Sci.

[54] Hamma T, Ferré-D’Amaré AR (2006). Pseudouridine synthases. Chem Biol.

[55] Pratt LA, Kolter R (1998). Genetic analysis of *Escherichia coli* biofilm formation: roles of flagella, motility, chemotaxis and type I pili. Mol Microbiol.

[56] Wang F, Deng L, Huang F, Wang Z, Lu Q (2020). Flagellar motility is critical for *Salmonella enterica* serovar Typhimurium biofilm development. Front Microbiol.

[57] Shimada T, Bridier A, Briandet R, Ishihama A (2011). Novel roles of LeuO in transcription regulation of *E. coli* genome: antagonistic interplay with the universal silencer H-NS. Mol Microbiol.

[58] Kroner GM, Wolfe MB, Freddolino PL (2019). *Escherichia coli* Lrp regulates one-third of the genome via direct, cooperative, and indirect routes. J Bacteriol.

[59] Tramonti A, De Canio M, De Biase D (2008). GadX/GadW-dependent regulation of the *Escherichia coli* acid fitness island: transcriptional control at the *gadY*-*gadW* divergent promoters and identification of four novel 42 bp GadX/GadW-specific binding sites. Mol Microbiol.

[60] Eichelberger KR, Sepúlveda VE, Ford J, Selitsky SR, Mieczkowski PA (2020). Tn-seq analysis identifies genes important for *Yersinia pestis* adherence during primary pneumonic plague. mSphere.

[61] Yang X, Wang J, Feng Z, Zhang X, Wang X (2019). Relation of the *pdxB-usg-truA-dedA* operon and the *truA* gene to the intracellular survival of *Salmonella enterica* serovar Typhimurium. Int J Mol Sci.

[62] Wucher BR, Bartlett TM, Hoyos M, Papenfort K, Persat A (2019). *Vibrio cholerae* filamentation promotes chitin surface attachment at the expense of competition in biofilms. Proc Natl Acad Sci USA.

[63] Anwar N, Rouf SF, Römling U, Rhen M (2014). Modulation of biofilm-formation in *Salmonella enterica* serovar Typhimurium by the periplasmic DsbA/DsbB oxidoreductase system requires the GGDEF-EAL domain protein STM3615. PLoS One.

[64] Magnusson LU, Gummesson B, Joksimović P, Farewell A, Nyström TI (2007). Identical, independent, and opposing roles of ppGpp and DksA in *Escherichia coli*. J Bacteriol.

[65] Smith DR, Price JE, Burby PE, Blanco LP, Chamberlain J (2017). The production of curli amyloid fibers is deeply integrated into the biology of *Escherichia coli*. Biomolecules.

[66] Hengge R (2020). Linking bacterial growth, survival, and multicellularity – small signaling molecules as triggers and drivers. Curr Opin Microbiol.

[67] Garavaglia M, Rossi E, Landini P (2012). The pyrimidine nucleotide biosynthetic pathway modulates production of biofilm determinants in *Escherichia coli*. PLoS One.

[68] Cepas V, Ballén V, Gabasa Y, Ramírez M, López Y (2020). Transposon insertion in the purL gene induces biofilm depletion in *Escherichia coli* ATCC 25922. Pathogens.

[69] Pfiffer V, Sarenko O, Possling A, Hengge R (2019). Genetic dissection of *Escherichia coli*’s master diguanylate cyclase DgcE: role of the N-terminal MASE1 domain and direct signal input from a GTPase partner system. PLoS Genet.

[70] Pesavento C, Becker G, Sommerfeldt N, Possling A, Tschowri N (2008). Inverse regulatory coordination of motility and curli-mediated adhesion in *Escherichia coli*. Genes Dev.

[71] Fang X, Gomelsky M (2010). A post-translational, c-di-GMP-dependent mechanism regulating flagellar motility. Mol Microbiol.

[72] Wood TK, González Barrios AF, Herzberg M, Lee J (2006). Motility influences biofilm architecture in *Escherichia coli*. Appl Microbiol Biotechnol.

[73] Li S, Liang H, Wei Z, Bai H, Li M (2019). An osmoregulatory mechanism operating through OmpR and LrhA controls the motile-sessile switch in the plant growth-promoting bacterium *Pantoea alhagi*. Appl Environ Microbiol.

[74] Barrios AFG, Zuo R, Ren D, Wood TK (2006). Hha, YbaJ, and OmpA regulate *Escherichia coli* K12 biofilm formation and conjugation plasmids abolish motility. Biotechnol Bioeng.

[75] García-Contreras R, Zhang X-S, Kim Y, Wood TK (2008). Protein translation and cell death: the role of rare tRNAs in biofilm formation and in activating dormant phage killer genes. PLoS One.

[76] Sharma VK, Bearson BL (2013). Hha controls *Escherichia coli* O157:H7 biofilm formation by differential regulation of global transcriptional regulators FlhDC and CsgD. Appl Environ Microbiol.

[77] Prouty AM, Gunn JS (2003). Comparative analysis of *Salmonella enterica* serovar Typhimurium biofilm formation on gallstones and on glass. Infect Immun.

[78] Hammar M, Bian Z, Normark S (1996). Nucleator-dependent intercellular assembly of adhesive curli organelles in *Escherichia coli*. Proc Natl Acad Sci USA.

[79] Chauhan A, Sakamoto C, Ghigo J-M, Beloin C (2013). Did I pick the right colony? Pitfalls in the study of regulation of the phase variable antigen 43 adhesin. PLoS One.

[80] Danese PN, Pratt LA, Dove SL, Kolter R (2000). The outer membrane protein, antigen 43, mediates cell-to-cell interactions within *Escherichia coli* biofilms. Mol Microbiol.

[81] Samanta P, Clark ER, Knutson K, Horne SM, Prüß BM (2013). OmpR and RcsB abolish temporal and spatial changes in expression of *flhD* in *Escherichia coli* biofilm. BMC Microbiol.

