# Supplementary material for: Massively parallel transposon mutagenesis identifies temporally essential genes for biofilm formation in Escherichia coli
Source: Microb Genom. 2021 Nov 16;7(11):000673. doi: 10.1099/mgen.0.000673 (PMC8743551; doi:10.1099/mgen.0.000673)
Supplement: Supplementary material 1 [file mgen-7-0673-s001.pdf]

Supplementary Information

Supplementary figure 1: Mean insertion frequencies per gene for each time point. Coloured points show mean insertion frequencies per gene in biofilm conditions compared to planktonic conditions for each time point. Black points show insertion frequencies per gene compared between identical replicates and show the experimental error. Mean insertion frequencies combine all replicates with and without promoter induction with IPTG.

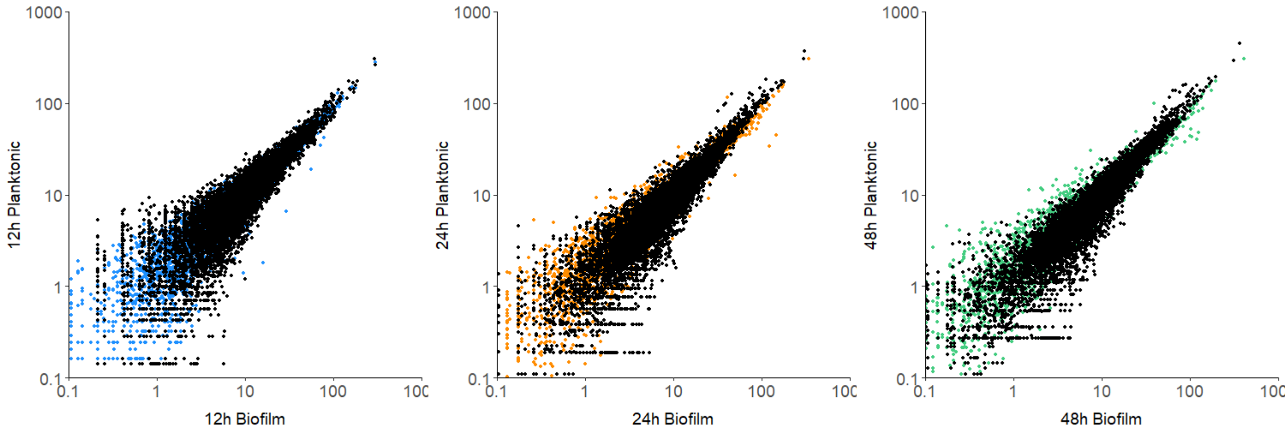

Supplementary table 1: Genes determined by TraDIS-*Xpress* to be important for biofilm formation in *E. coli*

**Pathway**	**Gene**	**Time point**	**Difference in insertions in biofilm condition relative to planktonic condition**		**Significantly different phenotype from wild type?**					**Ref**
			**Log fold change ***	**Observed change**	**Biomass**	**Aggregation**	**Curli production**	**Adhesion**	**Biofilm architecture**	
Cell division	*zapE*	48h	-3.4	Fewer insertions	No change	Increased	No change	Reduced	No change	1
c-di-GMP metabolism	*rcdA*	48h	-0.8	Fewer insertions	Reduced		Reduced			2
	pdeF	48h	-0.3	Fewer insertions						3
Curli biosynthesis	*csgC*	24h, 48h	-1.6 -0.6	Fewer insertions						4
	csgD	24h, 48h	-1.4	Increased expression beneficial at 24h, Fewer insertions at 48h	Reduced	Reduced	Reduced			
	csgE	12h, 48h	-2.5 -1.6	Fewer insertions	Reduced		Reduced			
	csgF	48h	-4.8	Fewer insertions						
DNA housekeeping	*dam*	24h	-3.9	Fewer insertions	No change	Reduced	No change			5
	maoP	24h	1.6	More insertions	Reduced	Reduced	Reduced	Reduced	Reduced density and biomass	6

Flagella-associated motility	*flhD*	24h,<br>48h	-3.9<br>-2.6	Increased expression beneficial	No change	No change	No change			7
	flhC	48h	-4.1	Fewer insertions	No change	Reduced	No change			
	flgD	24h	-3.0	Fewer insertions	No change	No change	No change			8
	fliE	48h	-4.7	Fewer insertions	No change	Reduced	No change			
	hdfR	12h,<br>24h	3.8<br>2.4	More insertions	Reduced	No change	Reduced			9
	lrhA	12h,<br>24h,<br>48h	2.0<br>3.2<br>2.3	More insertions	No change	Reduced	No change	Increased	Early microcolony formation, reduced in the mature biofilm	10
LPS	*wzzB*	48h	-1.4	Fewer insertions	Reduced		No change			11
Oxidised protein repair	*msrQ*	48h	-0.4	Fewer insertions	No change		No change			12
Protein modification	*dsbA*	12h,<br>24h	-0.7<br>-3.0	Fewer insertions	No change	Increased	Increased			13
Purine ribonucleotide biosynthesis	*purD*	48h	-4.3	Fewer insertions	Reduced	No change	Reduced	No change	Reduced microcolony formation	14
	purE	48h	-5.7	Fewer insertions	Reduced	Increased	Reduced			
	purH	48h	-3.2	Fewer insertions						

	purL	48h	-3.1	Fewer insertions						
rRNA methyltransferase	*rlmI*	12h	-3.8	Fewer insertions	Reduced	No change	No change			[15]
RNase III regulator	*ymdB*	24h, 48h	-0.5 -2.5	Fewer insertions	Reduced	Increased	No change			[16]
Sugar metabolism and transport	*sgbE*	48h	-2.5	Fewer insertions	No change		No change			[17]
Toxin-antitoxin system	*tomB*	12h, 24h, 48h	-0.5 -0.4 -1.6	Fewer insertions	Reduced	Reduced	Reduced	Increased	Early microcolony formation, reduced in the mature biofilm	[18]
Transcriptional regulators and signalling systems	*dksA*	12h, 24h	4.4 2.9	More insertions	Reduced	Reduced	Reduced	Increased	Reduced microcolony formation	[19-21]
	leuO	12h, 48h	-0.6	Increased expression beneficial at 12h, Fewer insertions at 48h	Reduced	Reduced	No change	No change	Reduced microcolony formation	[22]
	marR	12h	-4.1	Fewer insertions	Reduced	No change	No change			[23]

	ompR	24h,<br>48h	-0.8<br>-4.7	Fewer insertions	Reduced	Reduced	Reduced			24
	lrp	48h	-5.9	Fewer insertions	Reduced	Reduced	Reduced			25
	gadW	48h	-1.1	Fewer insertions	No change	No change	No change			26
	rcsC	48h	-2.9	Fewer insertions	Reduced		No change			27
Transmembrane transport, porins and channels	*mscL*	48h	-0.9	Fewer insertions						28
	tolC	48h	-2.9	Fewer insertions	No change	Reduced	No change			29
	ompF	48h	-2.7	Fewer insertions	Reduced		No change			24
	fadL	48h	-1.5	Fewer insertions						30
tRNA modification	*truA*	24h,<br>48h	-3.3<br>-5.9	Fewer insertions	No change	Increased	No change	No change	Increased filamentation after 24- and 48-hours growth	31
Type 1 fimbriae	*fimB*	12h,<br>24,<br>48h	-0.4<br>-1.3<br>-2.1	Fewer insertions and increased expression beneficial at all time points	No change	Reduced	No change			32
	fimE	12h,<br>24,<br>48h	1.5<br>3.3<br>2.6	More insertions	Reduced	Reduced	No change			
	fimC	48h	-1.3	Fewer insertions						33

	fimD	24h, 48h	-2.3 -2.1	Fewer insertions						[33]
Putative fimbrial-like protein	*ydeR*	48h	-2.4	Fewer insertions						[34]
Unknown	*yigZ*	12h	-2.8	Fewer insertions	No change	Increased	No change	No change	No change	[35]
	yebB	48h	-2.3	Fewer insertions						[36,37]
	yedN	48h	-1.7	Fewer insertions						[38]
	yjbL	48h	-2.8	Fewer insertions	Reduced		No change			[15]
	ykgJ	12h		Reduced expression beneficial (increased expression of antisense mRNA beneficial)	No change	Increased	No change	No change	Increased filamentation after 24- and 48-hours growth	[39]

* Log fold change is only shown for genes where there are differences in insertion frequency inside the coding region. Where the plot files generated by TraDIS-*Xpress* show a difference in insertion frequency between the biofilm and planktonic conditions upstream or downstream of a gene, log fold change cannot easily by quantified and therefore the effect has been described in the column titled ‘observed change’. Significant differences in insertion frequencies have been manually verified with the plot files generated by TraDIS-*Xpress*.

References

1 Marteyn, B. S. *et al.* ZapE is a novel cell division protein interacting with FtsZ and modulating the Z-ring dynamics. *mBio* **5**, e00022-00014, doi:10.1128/mBio.00022-14 (2014).

2 Pfiffer, V., Sarenko, O., Possling, A. & Hengge, R. Genetic dissection of *Escherichia coli*'s master diguanylate cyclase DgcE: Role of the N-terminal MASE1 domain and direct signal input from a GTPase partner system. *PLoS Genet.* **15**, e1008059, doi:10.1371/journal.pgen.1008059 (2019).

3 Lacey, M. M., Partridge, J. D. & Green, J. *Escherichia coli* K-12 YfgF is an anaerobic cyclic di-GMP phosphodiesterase with roles in cell surface remodelling and the oxidative stress response. *Microbiology* **156**, 2873-2886, doi:10.1099/mic.0.037887-0 (2010).

4 Barnhart, M. M. & Chapman, M. R. Curli Biogenesis and Function. *Annu. Rev. Microbiol.* **60**, 131-147, doi:10.1146/annurev.micro.60.080805.142106 (2006).

5 Szyf, M. *et al.* DNA methylation pattern is determined by the intracellular level of the methylase. *Proc. Natl. Acad. Sci. U. S. A.* **81**, 3278-3282, doi:10.1073/pnas.81.11.3278 (1984).

6 Valens, M., Thiel, A. & Boccard, F. The MaoP/*maoS* Site-Specific System Organizes the Ori Region of the *E. coli* Chromosome into a Macrodomain. *PLoS Genet.* **12**, e1006309-e1006309, doi:10.1371/journal.pgen.1006309 (2016).

7 Fitzgerald, D. M., Bonocora, R. P. & Wade, J. T. Comprehensive Mapping of the *Escherichia coli* Flagellar Regulatory Network. *PLoS Genet.* **10**, e1004649, doi:10.1371/journal.pgen.1004649 (2014).

8 Macnab, R. M. Genetics and biogenesis of bacterial flagella. *Annu. Rev. Genet.* **26**, 131-158, doi:10.1146/annurev.ge.26.120192.001023 (1992).

9 Ko, M. & Park, C. H-NS-Dependent regulation of flagellar synthesis is mediated by a LysR family protein. *J. Bacteriol.* **182**, 4670-4672, doi:10.1128/jb.182.16.4670-4672.2000 (2000).

10 Lehnen, D. *et al.* LrhA as a new transcriptional key regulator of flagella, motility and chemotaxis genes in *Escherichia coli*. *Mol. Microbiol.* **45**, 521-532, doi:10.1046/j.1365-2958.2002.03032.x (2002).

11 Stenberg, F. *et al.* Protein complexes of the *Escherichia coli* cell envelope. *J. Biol. Chem.* **280**, 34409-34419, doi:10.1074/jbc.M506479200 (2005).

12 Gennaris, A. *et al.* Repairing oxidized proteins in the bacterial envelope using respiratory chain electrons. *Nature* **528**, 409-412, doi:10.1038/nature15764 (2015).

13 Bardwell, J. C. Building bridges: disulphide bond formation in the cell. *Mol. Microbiol.* **14**, 199-205, doi:10.1111/j.1365-2958.1994.tb01281.x (1994).

14 Zhang, Y., Morar, M. & Ealick, S. E. Structural biology of the purine biosynthetic pathway. *Cellular and Molecular Life Sciences* **65**, 3699-3724, doi:10.1007/s00018-008-8295-8 (2008).

15 Herzberg, M., Kaye, I. K., Peti, W. & Wood, T. K. YdgG (TqsA) controls biofilm formation in *Escherichia coli* K-12 through autoinducer 2 transport. *J. Bacteriol.* **188**, 587-598, doi:10.1128/jb.188.2.587-598.2006 (2006).

16 Kim, T., Lee, J. & Kim, K.-s. *Escherichia coli* YmdB regulates biofilm formation independently of its role as an RNase III modulator. *BMC Microbiol.* **13**, 266-266, doi:10.1186/1471-2180-13-266 (2013).

17 Yew, W. S. & Gerlt, J. A. Utilization of L-ascorbate by *Escherichia coli* K-12: assignments of functions to products of the *yjf-sga* and *yia-sgb* operons. *J. Bacteriol.* **184**, 302-306, doi:10.1128/jb.184.1.302-306.2002 (2002).

18 Garcia-Contreras, R., Zhang, X. S., Kim, Y. & Wood, T. K. Protein translation and cell death: the role of rare tRNAs in biofilm formation and in activating dormant phage killer genes. *PLoS One* **3**, e2394, doi:10.1371/journal.pone.0002394 (2008).
19 Girard, M. E. *et al.* DksA and ppGpp Regulate the σS Stress Response by Activating Promoters for the Small RNA DsrA and the Anti-Adapter Protein IraP. *J. Bacteriol.* **200**, e00463-00417, doi:10.1128/jb.00463-17 (2018).
20 Lemke, J. J., Durfee, T. & Gourse, R. L. DksA and ppGpp directly regulate transcription of the *Escherichia coli* flagellar cascade. *Mol. Microbiol.* **74**, 1368-1379, doi:10.1111/j.1365-2958.2009.06939.x (2009).
21 Mallik, P., Paul, B. J., Rutherford, S. T., Gourse, R. L. & Osuna, R. DksA is required for growth phase-dependent regulation, growth rate-dependent control, and stringent control of *fis* expression in *Escherichia coli*. *J. Bacteriol.* **188**, 5775-5782, doi:10.1128/JB.00276-06 (2006).
22 Dillon, S. C. *et al.* LeuO is a global regulator of gene expression in *Salmonella enterica* serovar Typhimurium. *Mol. Microbiol.* **85**, 1072-1089, doi:10.1111/j.1365-2958.2012.08162.x (2012).
23 Alekshun, M. N. & Levy, S. B. Alteration of the Repressor Activity of MarR, the Negative Regulator of the *Escherichia coli marRAB* Locus, by Multiple Chemicals In Vitro. *J. Bacteriol.* **181**, 4669-4672 (1999).
24 Cai, S. J. & Inouye, M. EnvZ-OmpR interaction and osmoregulation in *Escherichia coli*. *J. Biol. Chem.* **277**, 24155-24161, doi:10.1074/jbc.M110715200 (2002).
25 Calvo, J. M. & Matthews, R. G. The leucine-responsive regulatory protein, a global regulator of metabolism in *Escherichia coli*. *Microbiol. Rev.* **58**, 466-490 (1994).
26 Tucker, D. L. *et al.* Genes of the GadX-GadW regulon in *Escherichia coli*. *J. Bacteriol.* **185**, 3190-3201, doi:10.1128/jb.185.10.3190-3201.2003 (2003).
27 Ferrières, L. & Clarke, D. J. The RcsC sensor kinase is required for normal biofilm formation in *Escherichia coli* K-12 and controls the expression of a regulon in response to growth on a solid surface. *Mol. Microbiol.* **50**, 1665-1682, doi:10.1046/j.1365-2958.2003.03815.x (2003).
28 Sukharev, S. I., Blount, P., Martinac, B., Blattner, F. R. & Kung, C. A large-conductance mechanosensitive channel in *E. coli* encoded by *mscL* alone. *Nature* **368**, 265-268, doi:10.1038/368265a0 (1994).
29 Morona, R., Manning, P. A. & Reeves, P. Identification and characterization of the TolC protein, an outer membrane protein from *Escherichia coli*. *J. Bacteriol.* **153**, 693-699 (1983).
30 Nunn, W. D. & Simons, R. W. Transport of long-chain fatty acids by *Escherichia coli*: mapping and characterization of mutants in the *fadL* gene. *Proc. Natl. Acad. Sci. U. S. A.* **75**, 3377-3381, doi:10.1073/pnas.75.7.3377 (1978).
31 Hamma, T. & Ferré-D'Amaré, A. R. Pseudouridine Synthases. *Chem. Biol.* **13**, 1125-1135, doi:https://doi.org/10.1016/j.chembiol.2006.09.009 (2006).
32 Klemm, P. Two regulatory *fim* genes, *fimB* and *fimE*, control the phase variation of type 1 fimbriae in *Escherichia coli*. *The EMBO Journal* **5**, 1389-1393, doi:10.1002/j.1460-2075.1986.tb04372.x (1986).
33 Allen, W. J., Phan, G. & Waksman, G. Pilus biogenesis at the outer membrane of Gram-negative bacterial pathogens. *Curr. Opin. Struct. Biol.* **22**, 500-506, doi:https://doi.org/10.1016/j.sbi.2012.02.001 (2012).
34 Da Re, S. *et al.* Identification of commensal *Escherichia coli* genes involved in biofilm resistance to pathogen colonization. *PLoS One* **8**, e61628, doi:10.1371/journal.pone.0061628 (2013).

35 Park, F. *et al.* Crystal structure of YIGZ, a conserved hypothetical protein from *Escherichia coli* k12 with a novel fold. *Proteins: Structure, Function, and Bioinformatics* **55**, 775-777, doi:10.1002/prot.20087 (2004).
36 Schurr, T., Nadir, E. & Margalit, H. Identification and characterization of *E.coli* ribosomal binding sites by free energy computation. *Nucleic Acids Res.* **21**, 4019-4023, doi:10.1093/nar/21.17.4019 (1993).
37 Alper, H. & Stephanopoulos, G. Uncovering the gene knockout landscape for improved lycopene production in *E. coli*. *Applied Microbiology and Biotechnology* **78**, 801-810, doi:10.1007/s00253-008-1373-x (2008).
38 Goodall, E. C. A. *et al.* The Essential Genome of *Escherichia coli* K-12. *mBio* **9**, e02096-02017, doi:10.1128/mBio.02096-17 (2018).
39 Kacharia, F. R., Millar, J. A. & Raghavan, R. Emergence of New sRNAs in Enteric Bacteria is Associated with Low Expression and Rapid Evolution. *J. Mol. Evol.* **84**, 204-213, doi:10.1007/s00239-017-9793-9 (2017).
